# Burnout and personality factors among surgical area nurses: a cross sectional multicentre study

**DOI:** 10.3389/fpubh.2024.1383735

**Published:** 2024-07-22

**Authors:** Almudena Velando-Soriano, Laura Pradas-Hernández, María José Membrive-Jiménez, Nora Suleiman-Martos, Jose L. Romero-Béjar, Emilia Inmaculada De La Fuente-Solana, Guillermo Arturo Cañadas-De La Fuente

**Affiliations:** ^1^San Cecilio Clinical University Hospital, Andalusian Health Service, Granada, Spain; ^2^Spanish Red Cross Nursing School, University of Sevilla, Sevilla, Spain; ^3^Faculty of Health Sciences, University of Granada, Granada, Spain; ^4^Department of Statistics and Operations Research, University of Granada, Granada, Spain; ^5^Brain, Mind and Behavior Research Center (CIMCYC), University of Granada, Campus Universitario de Cartuja, Granada, Spain

**Keywords:** medical-surgical nursing, personality, burnout—professional, nursing, occupational disease

## Abstract

**Objective:**

To determine levels of burnout among surgical area nurses in Andalusia (Spain), to identify the phase of burnout in each participant and to consider its relationship with sociodemographic, occupational variables and personality factors considered.

**Data source:**

Data were collected by means of questionary. All nurses working in the surgical area on the date of data collection participated in the study. Sociodemographic and related to work variables were addressed in the questionnaire. Symptoms of anxiety and depression were measured using the Educational-Clinical Questionnaire: Anxiety and Depression (CECAD). Psychological personality variables were assessed using the NEO Five-Factor Inventory (NEO-FFI), adapted for a Spanish population. Burnout was measured using the Maslach Burnout Inventory (MBI).

**Study design:**

Multicentre, cross-sectional, quantitative study carried out from August to October 2021.

**Data analysis:**

Descriptive analysis, Student’s *t*-test for independent samples, Pearson’s correlation and multiple linear regression were performed with SPSS 25.0.

**Data extraction methods:**

The study sample consisted of 214 surgical area nurses at 23 hospitals in Andalusia (Spain). Sociodemographic, occupational and personality variables were studied using the Maslach Burnout Inventory, the NEO Five-Factor Inventory (NEO-FFI) and the Educational-Clinical Questionnaire: Anxiety and Depression. The STROBE statement guidelines were applied.

**Principal findings:**

29.4% of the nurses in the sample presented high levels of emotional exhaustion, 25.7% suffered from depersonalization and 28% had low levels of personal accomplishment. These three dimensions were significantly correlated with the NEO-FFI subscales (neuroticism, agreeableness, openness, conscientiousness and extraversion), and with all the anxiety and depression items considered. Agreeableness was a statistically significant predictor (*p* < 0.001) for all three dimensions of burnout.

**Conclusion:**

Nurses in the surgical area present high levels of Burnout. There is evidence that relates Burnout to personality factors and socio-demographic variables.

## Introduction

Burnout mainly affects those whose work involves direct, constant contact with other people, as is the case of nurses, who are inevitably in close proximity with patients and their problems (physical, psychological, and social) and hence are especially vulnerable to Burnout syndrome (1–4). This syndrome, first described in the 1970s, has the following dimensions: emotional exhaustion (EE), characterized by feelings of physical and mental tiredness; depersonalization (D), related to the appearance of cynical and/or negative attitudes toward co-workers and patients; and low levels of personal accomplishment (PA) or negative self-perception related to job performance (5, 6). According to the model of Golembiewski et al. (7), burnout can be classified into eight phases. Cahoon and Rowney (8) grouped the eight phases into three, as follows: Mild burnout: phases I (low scores in all MBI subscales), II and III; Moderate burnout: phases IV and V; High burnout: phases VI, VII and VIII (high scores on all MBI scales).

Burnout can cause serious health problems (9), both physical (such as type 2 diabetes, hypercholesterolaemia and respiratory, heart and intestinal pathologies) and mental (insomnia, suicidal thoughts, anxiety and depression).

In addition to these problems, Burnout can affect work performance, provoking errors and poor performance, heightening patient morbidity and mortality (10). The World Health Organization (11) recognized Burnout Syndrome as a medical diagnosis in 2019 and describes it as an individual response to chronic stress.

Certain personal and external factors may be related to the appearance and development of Burnout (12, 13), including the area of work in which nurses are employed (14–16). Personality variables influence the appearance and development of burnout. A relationship has been shown between neuroticism and burnout, suggesting it as a predictor of the disorder. The greater neuroticism, the greater emotional exhaustion and less personal accomplishment. The other four traits have been shown in different studies to have a negative association with burnout, being protective factors (1, 17–21). Other psychological variables to highlight are anxiety and depression. Depression and burnout have been the subject of multiple studies, reaching the conclusion that burnout, when accompanied by feelings of inferiority, can lead to depression. Like depression, anxiety can also appear together with the syndrome, be a consequence or precede it (22).

## Background

Surgical nurses provide comprehensive care to patients from the time they are received (for scheduled or urgent attention) until their transfer from the resuscitation units to the corresponding ward. These nurses are responsible for the patients during the entire surgical process (23). Rising life expectancy among the general population has led to the increasing presence of diseases such as cancer and cardiovascular problems. In consequence, more interventions are being performed and surgical workloads have become heavier (24). However, during the COVID-19 pandemic, the number of operations performed, including emergency operations, decreased considerably (by up to 19%), due to patients’ fear of seeking emergency hospital attention. However, this situation lengthened surgical waiting lists, which increased pressures on surgical area staff when the worst effects of the pandemic subsided. This work overload also led to the development of Burnout (25).

It is important to highlight the specialization that is required in operating rooms. In many cases, workers do not have the required specialization, which results into a shortage of qualified personnel (26).

Surgical nurses are exposed to various factors that may be a risk for the development of burnout. Surgical area is a closed area, with sometimes excessive working hours, serious medical conditions of the patients and terrible consequences in the case of malpractice; In this environment, not the slightest error is expected, which requires high concentration and constant attention (27). In addition, there are biological, chemical and physical factors (exposure to disinfectants, chemicals, residual anesthetic gasses, sharp objects and blood pathogens, long hours standing and holding instruments and equipment during surgery) that can also influence development of burnout (28).

There is currently not much research available to determine the prevalence and impact of Burnout among nurses working in the surgical area, specifically. Most studies tend to analyze the Burnout experienced by surgical and medical nurses, reporting that a moderate-high level of Burnout is significantly associated with the nurse’s intention to abandon the profession (29, 30).

## Aims

The aims set out at the beginning of this study are: (1) to analyze the level of Burnout among nurses working in the surgical areas of the hospitals considered in Andalusia (Spain); (2) to estimate the phase of Burnout experienced in each participant, using Golembiewski’s model; (3) to examine the relationship between these phases of Burnout and the occupational, sociodemographic variables and personality factors addressed.

## Methods

### Design

Multicentre, cross-sectional, quantitative study.

### Data collection

The study was carried out from August to October 2021 with a convenience sampling. The study sample consisted of 214 surgical area nurses at 23 Andalusian Health Service hospitals, southern Spain (all eight provinces in the region were represented), who were working at that time. In total, supervisors within the surgical areas of the hospitals distributed 300 paper-format questionnaires. Of the 237 questionnaires returned, 214 were completed and subsequently analyzed. The mean age of the nurses was 43.98 years old, with a standard deviation of 9.01. 68.2% were female. According to official data of the Andalusian Health Service, 4,800 nurses are currently working in the surgical areas of its hospitals.

First, they were given written information about the study. All nurses working in the surgical area on the date of data collection participated in the study. Participation was voluntary and anonymous. After that the questionnaires were delivered to their workstations, and sufficient time was allowed for their completion. The questionnaire took approximately 45 min to complete. Members of the research team in collaboration with the Andalusian delegation of the Nursing Union collected all data.

### Variables and instruments

The following sociodemographic variables were addressed in the questionnaire: sex, age, marital status and number of children. Some work variables that have been related in the literature with Burnout are included. The variables related to work were: work shift (stable vs. rotating), on-call duty (yes vs. no) and length of service in the surgical area and in the nursing profession.

In addition to the above, psychological variables were considered, in conjunction with the MBI scale (31) adapted by Seisdedos (32), to measure Burnout syndrome. This instrument is composed of 22 items with a 7-point Likert scale ranging from 0 (Never) to 6 (Always). The test was evaluated according to the results obtained for each of the three dimensions of Burnout: EE (nine items), (five items) and low PA (eight items). High levels of EE and D and low ones of PA are indicative of Burnout. The cut-off points used to determine high levels of Burnout were > 24 for EE, >9 for D, and < 33 for low PA. The reliability of the questionnaire was determined by calculating the Cronbach’s alpha value for each dimension of the scale, with the following results: EE = 0.887; D = 0.692 and PA = 0.842.

Symptoms of anxiety and depression were measured using the CECAD Educational-Clinical Questionnaire (33). In addition to depression and anxiety, this instrument considers four clinical aspects (feelings of uselessness, irritability, problems related to thought and reasoning processes, and psychophysiological symptoms) considering the criteria of the Diagnostic and Statistical Manual of Mental Disorders (34). This questionnaire contains 50 items scored on a 5-point Likert scale. The study uses 45 items corresponding to the dimensions of anxiety and depression; 26 assess depression and 19 assess anxiety. The Cronbach’s alpha values for the CECAD scales of anxiety and depression were *α* = 0.905 and *α* = 0.924, respectively.

Psychological personality variables were assessed using the NEO Five-Factor Inventory (NEO-FFI), adapted for a Spanish population from The Diagnostic Criteria of DSM5 Reference Guide of the American Psychiatric Association (35). This instrument measures the main personality factors: neuroticism, extraversion, agreeableness, openness and conscientiousness. The NEO-FFI consists of 60 items (12 for each dimension) scored on a 5-point Likert scale. The reliability coefficients calculated were as follows: extraversion (*α* = 0.775), agreeableness (*α* = 0.644), conscientiousness (*α* = 0.787), openness to experience (*α* = 0.718) and neuroticism (*α* = 0.762).

### Data analysis

Descriptive statistics (mean, standard deviation, and maximum/minimum values) were analyzed from the quantitative variables. Frequencies and percentages were obtained for qualitative variables.

Student’s *t*-test for independent samples was applied to the qualitative variables from which the MBI dimensions were derived. Pearson’s correlation was used to determine the relationship between quantitative variables. Finally, multiple linear regression was performed for each dimension of Burnout (MBI) with all the variables. The presence of normal distribution and homoscedasticity was confirmed.

SPSS 25.0 was used for all analyzes (IBM, Armonk, NY, United States) statistical package.

Our analysis was conducted in line with the Strengthening the Reporting of Observational studies in Epidemiology (STROBE) checklist for cross-sectional studies (36).

### Ethical considerations

The study was conducted at all times in compliance with the provisions of the Declaration of Helsinki and was approved by Biomedical Research Ethics Portal in Andalusia (PEIBA) with reference number 1961-N-21 and Ethics Committee of the University of Granada with reference number 3892/CEIH/2023 (37). Moreover, all study data were processed in accordance with Organic Law 3/2018 on Data Protection and the Guarantee of Digital Rights (38).

## Results

### Sociodemographic data

Table 1 details the descriptive statistical data obtained from the qualitative variables. Among the noteworthy findings made, 68.2% of participants were female, 71.8% were married, 74.6% had one or more children, 56.5% worked rotating shifts and 75.9% did not perform on-call duties.

**Table 1:** Descriptive data of the qualitative variables.

Variable	*n* (%)
**Sex**	
Male	68 (31.8)
Female	146 (68.2)
**Work shift**	
Fixed—morning	87 (40.7)
Fixed—evening	2 (0.9)
Fixed—night	4 (1.9)
Rotating	121 (56.5)
**On-call duties**	
Yes	51 (24.1)
No	161 (75.9)
**Marital status**	
Single	43 (20.2)
Married	153 (71.8)
Separated	14 (6.6)
Divorced	3 (1.4)
Widowed	0 (0)
**Children**	
None	54 (25.4)
One	34 (16.0)
Two	96 (45.0)
Three or more	29 (13.6)

Table 2 shows the descriptive analysis of the socio-demographic variables and the dimensions of the NEO-FFI, the CECAD, and the MBI.

**Table 2:** Descriptive data of the quantitative variables.

Variable	Mean (SD)	Minimum–maximum
Age	43.98 (9.01)	23–63
Length of service in the surgical area (months)	136.38 (116.66)	2–600
Length of service in the profession (months)	246.25 (106.19)	12–600
**NEO-FFI**		
Neuroticism	28.11 (7.26)	12–50
Extraversion	42.64 (7.03)	21–59
Openness	38.89 (7.00)	21–56
Agreeableness	45.78 (6.22)	23–60
Conscientiousness	47.70 (6.12)	24–60
**CECAD**		
Anxiety	37.23 (11.14)	19–76
Depression	51.05 (14.69)	26–108
**MBI**		
Emotional exhaustion	19.69 (11.49)	0–54
Depersonalization	6.25 (5.57)	0–24
Personal accomplishment	36.70 (8.42)	0–48

NEO-FFI, revised NEO five factor inventory; CECAD, the educational-clinical questionnaire: anxiety and depression; MBI, maslach burnout inventory.

### Estimated prevalence and levels of burnout

To determine levels of Burnout, participants were classified according to their levels (low, medium, or high) in each of the dimensions considered, according to the MBI cut-off points (as adapted for the Spanish population), with the following results: for EE, 38.8% had a low level, 31.8% an medium level and 29.4% a high level. For D, the corresponding levels were: low 39.7%, medium 34.6% and high 25.7%. Finally for PA, 28% had a low level, 31.8% a medium level and 40.2% had a high level.

### Determining the phase of burnout syndrome

Golembiewski’s model (7, 39) was used to classify participants according to levels and phases. Low levels of Burnout comprise phases I, II, and III, medium levels comprise phases IV and V and high levels comprise phases VI, VII, and VIII. According to this model, 33.2% of our participants presented high levels of Burnout (Table 3).

**Table 3:** Burnout prevalence according to the phases of the Golembiewski model.

Phase	I	II	III	IV	V	VI	VII	VIII
EE	L	L	L	L	H	H	H	H
D	L	H	L	H	L	H	L	H
PA	L	L	H	H	L	L	H	H
*n*	63	24	25	9	22	16	18	37
(%)	29.4	11.2	11.7	4.2	10.3	7.5	8.4	17.3

EE, emotional exhaustion; D, depersonalization; PA, personal accomplishment; L, low; H, high.

### Mean difference analysis between levels of burnout and the sociodemographic and organizational factors considered

The mean values for each MBI dimension were compared in relation to the sociodemographic and occupational factors considered – gender, work shift, on-call duties, marital status and children. Significant differences were observed between men and women for D (*t* = 3.053, *p* < 0.01, *d* = 0.5). Men had the highest values in this dimension.

### Explanatory models between burnout levels and psychological factors

A linear correlation analysis was performed of the MBI dimensions, the NEO-FFI subscales and the CECAD depression and anxiety scores. All the Burnout dimensions presented statistically significant correlations with all the NEO-FFI subscales and also with all the CEDAD anxiety and depression items (Table 4).

**Table 4:** Coefficient of correlation between psychological variables and burnout.

	EE	D	PA
**NEO-FFI**			
Neuroticism	0.588^**^	0.414^**^	−0.464^**^
Extraversion	−0.390^**^	−0.287^**^	0.513^**^
Conscientiousness	−0.242^**^	−0.379^**^	0.634^**^
Agreeableness	−0.362^**^	−0.433^**^	0.530^**^
Openness	−0.161^*^	−0.193^**^	0.346^**^
**CECAD**			
Depression	0.617^**^	0.545^**^	−0.485^**^
Anxiety	0.585^**^	0.495^**^	−0.460^**^

EE, emotional exhaustion; D, depersonalization; PA, Personal accomplishment. ^*^p < 0.05, ^**^p < 0.001.

### Multiple linear regression for burnout dimensions

A multiple linear regression model was obtained for Burnout dimensions. This model explains 47.3% of the variability (*R*^2^ = 0.473) of EE. In this dimension, the following variables were statistically significant predictors: depression (*B* = 0.206; *p* < 0.001), neuroticism (*B* = 0.478; *p* < 0.001), agreeableness (*B* = −0.304; *p* < 0.001) and conscientiousness (*B* = 0.298, *p* < 0.01). For D, the goodness of fit presented a value of R^2^ = 0.403. For this dimension, the following variables were statistically significant predictors: sex (*B* = −2.411; *p* < 0.001), depression (*B* = 0.127; *p* < 0.01) and agreeableness (*B* = −0.173; *p* < 0.01). Finally, the model explains 56.1% of the variability in PA (*R*^2^ = 0.561). In this case, the following variables were statistically significant predictors: neuroticism (*B* = −0.169; *p* < 0.05), conscientiousness (*B* = 0.490; *p* < 0.001), extraversion (*B* = 0.244; *p* < 0.001) and agreeableness (*B* = 0.293; *p* < 0.001) (Table 5).

**Table 5:** Multiple linear regression.

	B	Standard error	Beta	*t*	*p*-value
**EE (*R***^2^ **= 0.473)**					
Neuroticism	0.478	0.112	0.302	4.252	<0.001
Agreeableness	−0.304	0.109	−0.165	−2.777	<0.01
Conscientiousness	0.298	0.118	0.159	2.523	<0.05
Depression	0.206	0.086	0.264	2.395	<0.05
**D (*R***^2^ **= 0.403)**					
Sex	−2.411	0.667	−0.202	−3.615	<0.001
Agreeableness	−0.173	0.057	−0.193	−3.018	<0.05
Depression	0.127	0.045	0.335	2.849	<0.05
**PA (*R***^2^ **= 0.561)**					
Neuroticism	−0.169	0.076	−0.146	−2.224	<0.05
Agreeableness	0.293	0.074	0.217	3.939	<0.001
Conscientiousness	0.490	0.079	0.356	6.174	<0.001
Extraversion	0.244	0.069	0.204	3.536	<0.001

EE, emotional exhaustion; D, depersonalization; PA, personal accomplishment; B, estimated parameter; t, Student’s *t*-test. Only the significant variables in the multiple regression model are displayed.

## Discussion

According to our analysis of the data obtained and responding to the stated objectives, to determine levels of burnout among surgical area nurses in Andalusia (Spain) and to identify the phase of burnout in each participant, we found that the 17.3% of the nurses who working in the surgical areas of the Andalusian Health Service hospitals present high levels of Burnout. This finding is in line with a similar study conducted in China, which reported that 24% of operating room nurses suffered from Burnout (29). However, it differs from another study, also carried out in China, according to which only 6.06% of nurses presented a high rate of Burnout (40). In our study, the following values were obtained for the prevalence of each dimension of Burnout: 29.1% of participants had a high level of EE, 24.9% a high level of D, and 47.5% low PA. These values are lower than those found in a smaller sample of nurses who provided perioperative attention at a hospital in Barcelona. In this case, 43% of the nurses suffered from high EE, 21% had high D and 53% had low PA (41). In another study, of Burnout among surgical medical personnel, only 8% of nurses presented low PA, but values for EE and D were higher than those of our study, at 41 and 59%, respectively (30). This variation may be due to the differences between the health systems considered (in Spain and the United States, respectively).

The prevalence of EE obtained in our analysis is similar to that reported for nurses working in pediatric and gynecology services (22 and 17%, respectively), and lower than that of those in accident and emergency services (31%) and among nurses employed in management positions (29%). For D, nurses working in the surgical area present higher values than those in pediatrics and gynecology (18.5 and 16.6%, respectively), while the highest values reported are for nurses in the emergency services (36%). However, with regard to low PA, only in the gynecology and obstetrics department were higher results than ours obtained (55.1%) (2, 14, 42).

Among the studies conducted, in response to the last objective raised, to consider its relationship with sociodemographic, occupational variables and personality factors considered, one factor that was observed by all was the presence of work overload and poor working conditions (43), resulting in decreased patient safety and quality of care, greater patient mortality and high levels of Burnout among healthcare personnel (44, 45).

Single and divorced men are more likely than women and married men to have high levels of Burnout, especially of D, expressed via negative attitudes and conflicts with co-workers (46), worsening interpersonal relationships and service quality (1, 10).

If we take into account psychological and personality variables, we can establish a Burnout risk profile. In our study, a statistically significant difference was found between depression, EE and D, corroborating previous research findings according to which depressive symptoms are associated with increased EE and D. Moreover, persons experiencing physical and psychological exhaustion are more likely to develop depression (47). Studies have also observed a positive relationship between EE and neuroticism, possibly due to the stress arising from work overload and from the specialized skills required to work in the operating room (40, 48). In addition, an inverse relationship has been detected between D and agreeableness; teamwork in the surgical area is essential, and one aspect of agreeableness is the willingness to help other people (48). PA bears a significant positive relationship with agreeableness, conscientiousness and extraversion, while it is inversely related with neuroticism (2). The fact that a high percentage of surgical area nurses present low PA may be due to organizational factors, such as inadequate supervision and support by the nursing manager, insufficient human and other resources, sub-optimum care quality provided or an excessive workload (30, 41).

### Applications to clinical practice

In developing strategies for improved healthcare, managers and policymakers should seek to prevent or reduce the presence of Burnout syndrome. The identification of risk groups, according to personality traits, would facilitate the early detection of Burnout and guide the necessary action to alleviate its impact on surgical area nurses. It would be very useful to conduct longitudinal studies to analyze the evolution of Burnout for future treatment protocols. In this respect, researchers should consider interventions that might prevent or reduce Burnout, such as meditation and the development of strategies for coping and enhancing resilience.

### Limitations

This study has some limitations. First, its cross-sectional design means that causal relationships cannot be established. In addition, although various psychological and sociodemographic variables were included in the analysis, it omits others that are also potentially relevant to the appearance, development and/or prevention of Burnout, including resilience, empathy and work-related factors such as the nurse-doctor relationship, the impact of hospital management and the degree of support and leadership shown by supervisors. Finally, this study was carried out in an area of Spain governed by a single health system and cannot necessarily be extrapolated to other countries with different health systems that may differ in their organization and way of working. In addition, the possible lack of representativeness of the sample is another limitation. In fact, convenience sampling has several limitations such as the appearance of data bias and the generation of inaccurate parameters.

## Conclusion

Our study shows that a third of the surgical area nurses considered present high levels of Burnout, especially of low PA. The psychological factors most strongly related to its appearance and development are neuroticism and depression. Among the sociodemographic variables analyzed, sex, and marital status were found predictors of Burnout, while agreeableness, conscientiousness and extraversion were protective factors.

### Relevance to clinical practice

In view of our finding that a large percentage of surgical area nurses present Burnout, it seems clear that more effective strategies are needed to protect the mental health of these workers. Both EE and D are significant factors, but the generalized presence of low PA is the most worrisome finding. This dimension is related to negative attitudes toward oneself and the work performed. It is associated with increased irritability, a loss of motivation and low self-esteem, all of which contribute to worsening patient care. By determining the psychological profile of nurses most susceptible to Burnout, steps could be taken to prevent or alleviate this condition at an early stage. Strategies to this end should be a priority concern for healthcare managers and policymakers. To support this endeavor, further studies are needed to better understand the personality and sociodemographic factors that influence the appearance of Burnout syndrome.

## Data availability statement

The raw data supporting the conclusions of this article will be made available by the authors, without undue reservation.

## Ethics statement

The studies involving humans were approved by the PEIBA (Biomedical Research Ethics Portal in Andalusia) with reference number 1961-N-21. The studies were conducted in accordance with the local legislation and institutional requirements. The participants provided their written informed consent to participate in this study.

## Author contributions

AV-S: Formal analysis, Visualization, Writing – original draft. LP-H: Conceptualization, Investigation, Writing – review & editing. MM-J: Conceptualization, Investigation, Writing – review & editing. NS-M: Data curation, Formal analysis, Resources, Visualization, Writing – review & editing. JR-B: Data curation, Formal analysis, Software, Visualization, Writing – review & editing. EF-S: Conceptualization, Funding acquisition, Investigation, Project administration, Visualization, Writing – review & editing. GC-D: Conceptualization, Funding acquisition, Investigation, Supervision, Visualization, Writing – review & editing.

## References

[1] Cañadas-De la Fuente GA Vargas C San Luis C García I Cañadas GR De la Fuente EI. Risk factors and prevalence of burnout syndrome in the nursing profession. Int J Nurs Stud. (2015) 52:240–9. doi: 10.1016/j.ijnurstu.2014.07.001 25062805

[2] De la Fuente-Solana EI Pradas-Hernández L González-Fernández CT Velando-Soriano A Martos-Cabrera MB Gómez-Urquiza JL. Burnout syndrome in Paediatric nurses: a multi-Centre study. Int J Environ Res Public Health. (2021) 18:1324. doi: 10.3390/ijerph18031324, PMID: 33535707 PMC7908244

[3] Maslach C Jackson SE. The measurement of experienced burnout. J Org Behav. (1981) 2:99–113. doi: 10.1002/job.4030020205

[4] Molina-Praena J Ramirez-Baena L Gómez-Urquiza J Cañadas G De la Fuente E Cañadas-De la Fuente G. Levels of burnout and risk factors in medical area nurses: a meta-analytic study. Int J Environ Res Public Health. (2018) 15:2800. doi: 10.3390/ijerph15122800, PMID: 30544672 PMC6313576

[5] Maslach C Schaufeli WB Leiter MP. Job burnout. Annu Rev Psychol. (2001) 52:397–422. doi: 10.1146/annurev.psych.52.1.397 11148311

[6] Schaufeli W Leiter M Maslach C. Burnout: 35 years of research and practice. IEEE Eng Manag Rev. (2010) 38:4–18. doi: 10.1109/emr.2010.5645750

[7] Golembiewski RT Munzenrider R Carter D. Phases of progressive burnout and their work site covariants: critical issues in OD research and praxis. J Appl Behav Sci. (1983) 19:461–81. doi: 10.1177/002188638301900408 10265310

[8] Cahoon AR Rowney JA. Managerial burnout: a comparison by sex and level of responsibility. J Hum Res Admin. (1984) 7:249–63.

[9] Salvagioni D.A.J. Melanda F.N. Mesas A.E. González A.D. Gabani F.L. Andrade S.M. De (2017). Physical, psychological and occupational consequences of job burnout: a systematic review of prospective studies. PLoS One 12:e0185781. doi: 10.1371/journal.pone.0185781, PMID: 28977041 PMC5627926

[10] Oliveira EG Garcia PC Citolino Filho CM de Nogueira LS. The influence of delayed admission to intensive care unit on mortality and nursing workload: a cohort study. Nurs Crit Care. (2018) 24:381–6. doi: 10.1111/nicc.12402, PMID: 30478867

[11] World Health Organization. Burn-out an ‘occupational phenomenon’: international classification of diseases World Health Organization (2019) Available at: https://www.who.int/news/item/28-05-2019-burn-out-an-occupational-phenomenon-international-classification-of-diseases.

[12] Cañadas-De la Fuente G Ortega E Ramirez-Baena L De la Fuente-Solana E Vargas C Gómez-Urquiza J. Gender, marital status, and children as risk factors for burnout in nurses: a Meta-analytic study. Int J Environ Res Public Health. (2018) 15:2102. doi: 10.3390/ijerph15102102, PMID: 30257449 PMC6209972

[13] Gómez-Urquiza JL de la Fuente-Solana EI Albendín-García L Vargas-Pecino C Ortega-Campos EM Cañadas-de la Fuente GA. Prevalence of burnout syndrome in emergency nurses: a meta-analysis. Crit Care Nurse. (2017) 37:e1–9. doi: 10.4037/ccn2017508, PMID: 28966203

[14] Gómez-Urquiza JL Monsalve-Reyes CS San Luis-Costas C Fernández-Castillo R Aguayo-Estremera R Cañadas-de la Fuente GA. Risk factors and burnout levels in primary care nurses: a systematic review. Atenc Prim. (2017) 49:77–85. doi: 10.1016/j.aprim.2016.05.004, PMID: 27363394 PMC6876264

[15] Ramírez-Elvira S Romero-Béjar JL Suleiman-Martos N Gómez-Urquiza JL Monsalve-Reyes C Cañadas-de la Fuente GA. Prevalence, risk factors and burnout levels in intensive care unit nurses: a systematic review and Meta-analysis. Int J Environ Res Public Health. (2021) 18:11432. doi: 10.3390/ijerph182111432, PMID: 34769948 PMC8583312

[16] Gómez-Urquiza JL Vargas C De la Fuente EI Fernández-Castillo R Cañadas-De la Fuente GA. Age as a risk factor for burnout syndrome in nursing professionals: a meta-analytic study. Res Nurs Health. (2016) 40:99–110. doi: 10.1002/nur.21774, PMID: 27862065

[17] Angelini G. Big five model personality traits and job burnout: a systematic literature review. BMC Psychol. (2023) 11:594–601. doi: 10.1186/s40359-023-01056-y, PMID: 36804929 PMC9938997

[18] De la Fuente-Solana EI Suleiman-Martos N Velando-Soriano A Cañadas-De la Fuente GR Herrera-Cabrerizo B Albendín-García L. Predictors of burnout of health professionals in the departments of maternity and gynaecology, and its association with personality factors: a multicentre study. J Clin Nurs. (2020) 30:207–16. doi: 10.1111/jocn.15541 33090612

[19] De la Fuente-Solana E Cañadas G Ramirez-Baena L Gómez-Urquiza J Ariza T Cañadas-De la Fuente G. An explanatory model of potential changes in burnout diagnosis according to personality factors in oncology nurses. Int J Environ Res Pub Health. (2019) 16:312. doi: 10.3390/ijerph16030312, PMID: 30678332 PMC6388253

[20] Fornés-Vives J García-Banda G Frias-Navarro D Pascual-Soler M. Longitudinal study predicting burnout in Spanish nurses: the role of neuroticism and emotional coping. Personal Individ Differ. (2019) 138:286–91. doi: 10.1016/j.paid.2018.10.014

[21] Ramirez-Baena L Ortega-Campos E Gomez-Urquiza J Cañadas-De la Fuente G De la Fuente-Solana E Cañadas-De la Fuente G. A multicentre study of burnout prevalence and related psychological variables in medical area hospital nurses. J Clin Med. (2019) 8:92. doi: 10.3390/jcm8010092, PMID: 30650557 PMC6351959

[22] Peiró JM Salvador A. Desencadenantes del estrés laboral. Madrid: Eudema (1999).

[23] Gilmour D Perioperative care Pudner R. Nursing the surgical patient. 3rd ed. Paris: Bailièrre Tindall (2010).

[24] Gensimore MM Maduro RS Morgan MK McGee GW Zimbro KS. The effect of nurse practice environment on retention and quality of care via burnout, work characteristics, and resilience. J Nurs Adm. (2020) 50:546–53. doi: 10.1097/nna.0000000000000932 32925666

[25] Kurihara H Marrano E Ceolin M Chiara O Faccincani R Bisagni P. Impact of lockdown on emergency general surgery during first 2020 COVID-19 outbreak. Eur J Trauma Emerg Surg. (2021) 47:677–82. doi: 10.1007/s00068-021-01691-3, PMID: 33944976 PMC8093909

[26] Cabana A García-Ceballos E García-García G Suárez AM Dávila-Ramírez R. El síndrome de Burnout en el personal de una unidad quirúrgica. Rev Méd Electr. (2009) 31

[27] Costa PT Mccrae RR Pamos A. Analysis of the dimensions and influential factors in occupational stress in the operating room staff of the teaching hospitals in Shahrekord, Iran. J. Clin. Nurs. (2017) 8:308–316.

[28] Teymoori E Zareiyan A Babajani-Vafsi S Laripour R. Viewpoint of operating room nurses about factors associated with the occupational burnout: A qualitative study. Front. Psychol. (2022) 13:947189. doi: 10.3389/fpsyg.2022.947189 36033007 PMC9403988

[29] Cheng L Yang J Li M Wang W. Mediating effect of coping style between empathy and burnout among Chinese nurses working in medical and surgical wards. Nurs Open. (2020) 7:1936–44. doi: 10.1002/nop2.584, PMID: 33072379 PMC7544859

[30] Phillips C. Relationships between workload perception, burnout, and intent to leave among medical–surgical nurses. Int J Evid Based Healthc. (2020) 18:265–73. doi: 10.1097/xeb.0000000000000220, PMID: 32141948

[31] Maslach C Jackson SE. Maslach burnout inventory: manual. 1st ed. Consulting Psychologists Press: Palo Alto (1981).

[32] Seisdedos-Cubero N. MBI: inventario ‘burnout’ de Maslach: síndrome del quemado por estrés laboral asistencial: manual. Madrid: TEA (1997).

[33] Lozano González L García Cueto E Manuel L. CECAD: cuestionario educativo-clínico: ansiedad y depresión. Madrid: TEA ediciones (2007).

[34] Costa PT Mccrae RR Pamos A Seisdedos-Cubero N. Inventario de personalidad neo revisado (NEO PI-R), inventario neo reducido de cinco factores (NEO-FFI): manual profesional. Madrid: TEA (2008).

[35] American Psychiatric Association. Desk reference to the diagnostic criteria from DSM-5-TM. Washington, DC: American Psychiatric Association (2022).

[36] Von Elm E Altman DG Egger M Pocock SJ Gøtzsche PC Vandenbroucke JP. The strengthening the reporting of observational studies in epidemiology (STROBE) statement: guidelines for reporting observational studies. PLoS Med. (2007) 4:e296. doi: 10.1371/journal.pmed.0040296, PMID: 17941714 PMC2020495

[37] World Medical Association. World medical association declaration of Helsinki. Ethical principles for medical research involving human subjects. Bull World Health Organ. (2001) 79:373. doi: 10.1001/jama.2013.281053 11357217 PMC2566407

[38] de Datos Personales P. Garantía de los derechos digitales. Ley Orgánica. (2018) 3:18.

[39] Golembiewski RT Munzenrider R. Phases of burnout. Westport, CT: Praeger (1988).

[40] Li X Jiang T Sun J Shi L Liu J. The relationship between occupational stress, job burnout and quality of life among surgical nurses in Xinjiang, China. BMC Nurs. (2021) 20:181. doi: 10.1186/s12912-021-00703-2, PMID: 34579710 PMC8477556

[41] Sillero A Zabalegui A. Organizational factors and burnout of perioperative nurses. Clin Prac Epidemiol Mental Health. (2018) 14:132–42. doi: 10.2174/1745017901814010132, PMID: 29997680 PMC5997854

[42] Membrive-Jiménez MJ Pradas-Hernández L Suleiman-Martos N Vargas-Román K Cañadas-De la Fuente GA Gomez-Urquiza JL. Burnout in nursing managers: a systematic review and Meta-analysis of related factors, levels and prevalence. Int J Environ Res Pub Health. (2020) 17:3983. doi: 10.3390/ijerph17113983, PMID: 32512738 PMC7312638

[43] Ślusarz R Filipska K Jabłońska R Królikowska A Szewczyk MT Wiśniewski A. Analysis of job burnout, satisfaction and work-related depression among neurological and neurosurgical nurses in Poland: a cross-sectional and multicentre study. Nurs Open. (2021) 9:1228–40. doi: 10.1002/nop2.1164, PMID: 34953049 PMC8859037

[44] Aiken LH Simonetti M Sloane DM Cerón C Soto P Bravo D. Hospital nurse staffing and patient outcomes in Chile: a multilevel cross-sectional study. The Lancet Glob Health. (2021) 9:e1145–53. doi: 10.1016/s2214-109x(21)00209-6, PMID: 34224669

[45] McHugh MD Aiken LH Windsor C Douglas C Yates P. Case for hospital nurse-to-patient ratio legislation in Queensland, Australia, hospitals: an observational study. BMJ Open. (2020) 10:e036264–7. doi: 10.1136/bmjopen-2019-036264, PMID: 32895270 PMC7476482

[46] Vahedian-Azimi A Hajiesmaeili M Kangasniemi M Fornés-Vives J Hunsucker RL Rahimibashar F. Effects of stress on critical care nurses: a national cross-sectional study. JIC. (2017) 34:311–22. doi: 10.1177/0885066617696853, PMID: 29277137

[47] Chen C Meier ST. Burnout and depression in nurses – a systematic review and meta-analysis. Int J Nurs Stud. (2021) 124:104099. doi: 10.1016/j.ijnurstu.2021.104099, PMID: 34715576

[48] De la Fuente-Solana EI Gómez-Urquiza JL Cañadas GR Albendín-García L Ortega-Campos E Cañadas-De la Fuente GA. Burnout and its relationship with personality factors in oncology nurses. Eur. J. Oncol. Nurs. (2017) 30:91–6. doi: 10.1016/j.ejon.2017.08.004 29031320

